# A standard photomap of ovarian nurse cell chromosomes and inversion polymorphism in *Anopheles beklemishevi*

**DOI:** 10.1186/s13071-018-2657-3

**Published:** 2018-03-27

**Authors:** Gleb N. Artemov, Mikhail I. Gordeev, Alina A. Kokhanenko, Anton V. Moskaev, Alena I. Velichevskaya, Vladimir N. Stegniy, Igor V. Sharakhov, Maria V. Sharakhova

**Affiliations:** 1 Laboratory of Ecology, Genetics and Environmental Protection, Tomsk State University, Tomsk, Russia; 2 Department of General Biology and Ecology, Moscow Regional State University, Moscow, Russia; 3 Virginia Tech, Department of Entomology, Fralin Life Science Institute, Blacksburg, VA USA

**Keywords:** *Anopheles*, Mosquito, Cytogenetic map, Inversion polymorphism, Physical mapping

## Abstract

**Background:**

*Anopheles beklemishevi* is a member of the Maculipennis group of malaria mosquitoes that has the most northern distribution among other members of the group. Although a cytogenetic map for the larval salivary gland chromosomes of this species has been developed, a high-quality standard cytogenetic photomap that enables genomics and population genetics studies of this mosquito at the adult stage is still lacking.

**Methods:**

In this study, a cytogenetic map for the polytene chromosomes of *An. beklemishevi* from ovarian nurse cells was developed using high-resolution digital imaging from field collected mosquitoes. PCR-amplified DNA probes for fluorescence *in situ* hybridization (FISH) were designed based on the genome of *An. atroparvus.* The DNA probe obtained by microdissection procedures from the breakpoint region was labelled in a DOP-PCR reaction. Population analysis was performed on 371 specimens collected in 18 locations.

**Results:**

We report the development of a high-quality standard photomap for the polytene chromosomes from ovarian nurse cells of *An. beklemishevi.* To confirm the suitability of the map for physical mapping, several PCR-amplified probes were mapped to the chromosomes of *An. beklemishevi* using FISH. In addition, we identified and mapped DNA probes to flanking regions of the breakpoints of two inversions on chromosome X of this species. Inversion polymorphism was determined in 13 geographically distant populations of *An. beklemishevi*. Four polymorphic inversions were detected. The positions of common chromosomal inversions were indicated on the map.

**Conclusions:**

The study constructed a standard photomap for ovarian nurse cell chromosomes of *An. beklemishevi* and tested its suitability for physical genome mapping and population studies. Cytogenetic analysis determined inversion polymorphism in natural populations of *An. beklemishevi* related to this species’ adaptation.

**Electronic supplementary material:**

The online version of this article (10.1186/s13071-018-2657-3) contains supplementary material, which is available to authorized users.

## Background

The malaria mosquito *Anopheles beklemishevi* Stegniy & Kabanova, 1976 [1] is a member of the Maculipennis group [2]. This group of mosquitoes has a wide distribution range in Eurasia and North America. Among members of the group, *An. beklemisevi* has the most northern distribution in Eurasia [3]. It occupies the territory from the East coast of the Baltic Sea to the basin of the Lena River and from the forest-tundra zone to the Altai and Sayan Mountain systems. The northern border of this species distribution in Russia lies to the North of the Arctic Circle. The geographical range of *An. beklemishevi* in the North substantially overlaps with the distribution of another Palearctic member of the Maculipennis group, *An. messeae* [4], which has wider distribution in western and southern Eurasia. As the most widespread mosquito in Eurasia, *An. messeae* is considered a dominant malaria vector in Eurasia [5]. Although *An. beklemishevi* is recognized as a malaria vector in Russia and Europe [6], it does not play an important role in malaria transmission because of its exophylic behavior and ecological preferences to high altitudes and swampy territories [3]. In fact, the ability of *An. beklemishevi* to transmit malaria has never been studied experimentally [7].

To date, only one cytogenetic map based on polytene chromosomes from the salivary glands has been developed for *An. beklemishevi* [8]. Images of chromosomes stained with lacto-aceto-orsein were obtained using photo films. According to the map, the polytene chromosomal complement of *An. beklemishevi* consists of three chromosomes with five chromosomal arms, which is the same as in other *Anopheles* species [9]. The X chromosome contains one arm, and both autosomes contain two chromosomal arms. Chromosomes on the map were numbered in the order of increasing size as in the nomenclature developed before [10]. Thus, the biggest autosome is chromosome 3. Chromosomes were subdivided into 39 regions in accordance with other chromosomal maps developed for the species from the Maculipennis group [11]. Landmarks for the chromosomal arm recognition were described in detail [8].

The cytogenetic map in the original study [8] has been successfully used to investigate inversion polymorphism in *An. beklemishevi.* Twelve polymorphic inversions have been identified in 1000 mosquito specimens from 15 populations distributed over the entire geographic range of the species. Five inversions were found on the longest chromosome arm 3R. Three inversions were detected on the 2R arm. Two inversions were observed on sex chromosome X, and only one inversion was detected in each of the 2L and 3L arms. Only two inversions, which are on the X chromosome, were highly polymorphic. Two individuals were homozygotes for one of the inversions on the X chromosome. In general, inversion polymorphism in *An. beklemishevi* remains understudied in comparison with *An. messeae* [12–19]. Moreover, because of the absence of a standard photomap for ovarian nurse cell chromosomes, the adult stage was excluded from the inversion polymorphism analysis.

In this study, we developed a high-quality standard cytogenetic photomap for ovarian nurse cell chromosomes of *An. beklemishevi*. We mapped four DNA probes amplified by polymerase chain reaction (PCR-amplified) on X, 2R, 3R, and 3L chromosome arms of *An. beklemishevi.* We also mapped the X chromosome inversion breakpoints using microdissected DNA probes. The original salivary gland map [8] and newly developed ovarian nurse cell map were used for the analysis of the inversion polymorphism at larval and adult stages, respectively. Thirteen distantly located populations of *An. beklemishevi* were analyzed and compared. Our study has demonstrated that a standard photomap of ovarian nurse cell chromosomes can be successfully utilized for physical genome mapping and for population genetic studies of the inversion polymorphism in *An. beklemishevi*.

## Methods

### Mosquito collection and ovary preservation

For the cytogenetic map development, *An. beklemishevi* mosquitoes were collected from a natural population in Chainsk (Tomsk region, Russia) in July and August of 2015 and in June of 2016 (Table 1). Ovaries of half-gravid females were dissected and fixed in Carnoy’s solution (3:1 ethanol: glacial acetic acid by volume). Ovaries were preserved in Carnoy’s solution from 24 h up to 1 month at -20 °C. For the inversion polymorphism analysis, mosquitoes were collected from 13 distant localities in Eurasia (Table 1). We studied a total of 371 mosquitoes from 18 collections comprised of 110 adults and 261 larvae. Collections in two populations, Kolarovo and Teguldet in Western Siberia, were conducted repeatedly during 3 and 4 years, respectively. The latter specimens were fixed in Carnoy’s solution and stored at 5 °C.

**Table 1:** Chromosomal polymorphism in populations of *An. beklemishevi*. Frequencies of inversion X1 and X2 were determined for females

Locality(coordinates)	Collection date	Number		Frequency of inversions			
		Total	Females	X1	X2	3R1	3R3
Kolarovo (56°20’N, 84°56′E)	07/04/1983	23L	8L	0	0.0625	0	0
Kolarovo (56°20’N, 84°56′E)	07/05/2004	6A	6A	0	0	0	0
Kolarovo (56°20’N, 84°56′E)	07/28/2005	2A	2A	0	0	0	0
Teguldet (57°18’N, 88°10′E)	08/05/2003	6A	6A	0	0	0	0
Teguldet (57°18’N, 88°10′E)	07/12/2005	27A	27A	0	0.1111	0	0
Teguldet (57°18’N, 88°10′E)	07/13/2006	4A	4A	0	0	0	0
Teguldet (57°18’N, 88°10′E)	08/01/2007	8A	8A	0	0	0	0
Artybash (51°47’N, 87°15′E)	07/26/2007	25L	13L	0	0.2308	0	0
Teletskoe Lake, Samysh River (51°45’N, 87°22′E)	07/27/2007	14L	9L	0	0.1667	0	0
Teletskoe Lake, Koldor River (51°47’N, 87°43′E)	07/28/2007	12L	5L	0	0	0	0
Teletskoe Lake, Kamga River (51°20’N, 87°47′E)	07/29/2007	15L	7L	0	0	0	0
Dvorets (57°56’N, 32°60′E)	06/01/2009	36L	17L	0	0.3236	0	0
Segezha (63°45’N, 34°46′E)	08/16/2010	64L	36L	0	0.1389	0	0
Belomorsk (64°31’N, 34°46′E)	08/14/2010	36L	20L	0.0750	0.0500	0.0417	0.0139
Dmitrovskiy Pogost (55°18’N, 39°50′E)	07/23/2015	27L	13L	0	0.1154	0	0
Parykino (39°23’N, 55°17′E)	07/24/2015	4L	3L	0	0.3333	0	0
Gzhel (55°36’N, 38°26′E)	08/20/2015	5L	4L	0	0.1250	0	0
Chainsk (57°55’N, 82°36′E)	06/22/2016	57A	57A	0.0088	0.1404	0	0

*Abbreviations*: *L* larvae, *A* adult females

### Chromosome preparation and species identification

For one preparation of ovarian nurse cell chromosomes, approximately a third part of a single ovary from one pair was taken. It is important to avoid having an excess of tissue on the slide for obtaining high-quality chromosome spreads. Ovaries were held for 5 min in a drop of 50% propionic acid, macerated and squashed. The quality of preparations was examined under an AxioImager A1 microscope (Carl Zeiss, OPTEC LLC, Novosibirsk, Russia). High-quality preparations were frozen in liquid nitrogen, and coverslips were removed. Preparations were dehydrated in an ethanol series (50%, 70%, 90%, and 100%) and air dried. Identification of species was conducted based on chromosome banding pattern [8, 20] and heterochromatin morphology [21]. Chromosomal preparations from salivary glands of 4th instar larvae were performed as described earlier [22]. Salivary glands were stained in 2% lacto-acetic-orcein for 45 min and squashed in 45% acetic acid. Karyotyping of polytene chromosomes was conducted using a Nikon, Eclipse E200 microscope (Nikon, BioVitrum, Moscow, Russia).

### Chromosome map development

Chromosome images were observed using an AxioImager A1 microscope (Carl Zeiss) with an attached CCD camera MRc5 in phase contrast using an AxioVision version 4.7.1 software (Carl Zeiss). For the chromosome map development, about 250 images of well-polytenized and well-spread chromosomes were obtained. Images were combined, straightened, shaped, and cropped using Adobe-Photoshop CS2 software. Chromosome nomenclature was generally adopted from the previously published chromosome map for salivary glands of *An. beklemishevi* [8, 20].

### Microdissection and DOP-PCR

Microdissection of region 2BC from the X chromosome of the ovarian nurse cells and subsequent DOP-PCR (Degenerate Oligonucleotide Primers PCR) were performed accordingly to the previously published protocol [23]. Microdissection by glass needles was performed from air-dried squashed preparations of ovarian nurse cell polytene chromosomes of *An. beklemishevi*. A micro-dissected DNA material was amplified with DOP primers to achieve the necessary amount of the region-specific DNA-probe. The DNA-probe was labeled in an additional PCR reaction in the presence of a fluorescently labelled nucleotide. The boundaries of the microdissected regions were chosen to represent the expected breakpoints of the polymorphic inversion.

### Gene-specific PCR

Gene-specific primers were designed to amplify four unique exon sequences using PRIMER-BLAST software available at NCBI [24]. Genes from different chromosome arms of *An. beklemishevi* were selected based on similarity with the *An. atroparvus* mapped genome [25]. The primer design was based on gene annotations from the AatrE1 genome assembly available at VectorBase [26]. PCR was performed in the presence of 1× PCR buffer (SibEnzyme Ltd., Novosibirsk, Russia), 2.5 mM MgCl2 (SibEnzyme Ltd., Novosibirsk, Russia), 0.2 mM dNTP (Thermo Fisher Scientific, Waltham, MA, USA), and 0.02 u/μl Taq Polymerase (SibEnzyme Ltd.).

### DNA probe labelling and fluorescence *in situ* hybridization

The DNA probe obtained by microdissection procedures was labelled in a DOP-PCR reaction in the presence of TAMRA-5-dUTP as described earlier [23]. Fragments obtained by gene-specific PCR were labelled using a Random Primer Labelling protocol: 25 μl of labelling reaction contained 50 ng DNA, 1× Klenow buffer (Thermo Fisher Scientific), 44 ng/μl Exo-Resistant Random Primer (Thermo Fisher Scientific) 0.1 mM dATP, dGTP, dCTP, and 0.015 mM dTTP, 0.016 mM TAMRA-5-dUTP, or Biotin-11-dUTP (Biosan, Novosibirsk, Russia), and 5 U of Klenow fragment (Thermo Fisher Scientific TM) in a PCR tube. The required amounts of DNA, Klenow buffer, and Random Primers were mixed, brought up to 12 μl with water, and heated at 95 °C for 5 min in a thermocycler. The solution was chilled on ice, and appropriate amounts of nucleotides, Klenow fragment, and water were added to reach 25 μl. The reaction mix was incubated at 37 °C for 18 h. Fluorescence *in situ* hybridization (FISH) was performed using a previously described standard protocol [25, 27].

### Statistical analysis

Frequencies of inversions were calculated as the fraction of all chromosomes in a population that carry the inversion. Chi-square test was used to examine whether inversion genotype frequencies were in Hardy-Weinberg equilibrium using an interactive calculation tool [28]. A *P*-value above 0.05 indicated that there are no significant differences between observed inversion frequencies in natural populations and expected inversion frequencies following the Hardy-Weinberg law (Additional file 1: Table S1).

## Results

### A cytogenetic map for *An. beklemishevi*

**Fig. 1:**
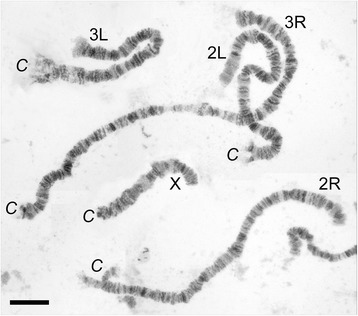
The chromosome complement in ovarian nurse cells of *An. beklemishevi*. Chromosome arms are shown as X, 2R, 2L, 3R and 3L. Pericentromeric regions are labeled as C. *Scale-bar*: 20 μm

We developed a high-resolution map for polytene chromosomes from ovarian nurse cells of *An. beklemishevi.* As shown before [8], the chromosome number in this species equals 3 as in other species of the genus *Anopheles* [10]. The polytene chromosome complement consists of 5 chromosome arms: 4 autosomal arms and one X chromosome arm (Fig. 1). Autosomal arms usually are separated from each other on a preparation because of fragile connections between the pericentromeric regions. The X chromosome is the shortest, and the 3R arm is the longest. The arm proportions (Table 2) are similar to those of chromosomes in salivary gland cells of other species of the Maculipennis group [25]. Interestingly, the banding patterns of ovarian nurse cell polytene chromosomes in *An. beklemishevi* are better quality than the banding patterns in other species in the Maculipennis group. The chromosomes have sharp band-interband borders and distinguishable thin bands. This feature differentiates *An. beklemishevi* from other Palearctic members of the Maculipennis group, such as *An. atroparvus* [25].

**Table 2:** Measurements of polytene chromosomes from *An. beklemishevi* ovarian nurse cells

	X	2	3
Average length (μm)	133	711	759
Relative length (%)	8.4	44.3	47.4
Centromere position (%)	na	48.8	38.0

**Fig. 2:**
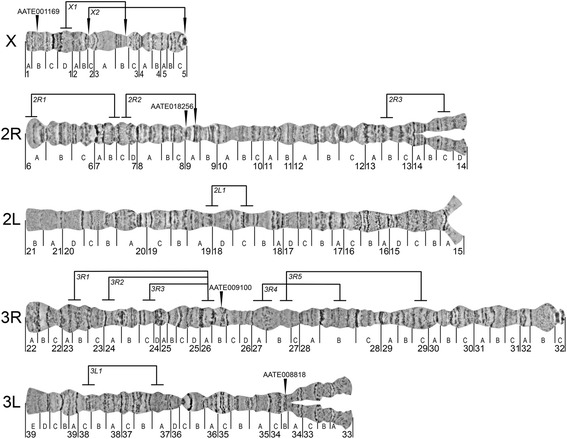
A standard cytogenetic photomap of ovarian nurse cell chromosomes of *Anopheles beklemishevi.* Chromosome arms are shown as X, 2R, 2L, 3R, and 3L. The vertical lines below the chromosomes indicate the boundaries of numbered and lettered divisions and subdivisions, respectively. Vertical arrowheads above the chromosomes indicate the locations of DNA probes of the four *An. atroparvus* genes orthologues. Brackets show inversions. Brackets with arrows indicate the exact positions of the inversions on X chromosome

The chromosome map of *An. beklemishevi* is divided into 39 numbered divisions and 122 lettered subdivisions (Fig. 2). Although division borders and nomenclature were adopted from the previously developed salivary gland chromosome map of *An. beklemishevi* [8], we reversed the order of lettered subdivisions in arms 2L and 3L to follow the order of numbered divisions.

Chromosomes in *An. beklemishevi* have well-distinguishable landmarks that simplify their identification. The sex chromosome X, the shortest chromosome, has a round pericentric heterochromatin band, usually followed by a neighboring thin band. In the middle of the chromosome, in region 2BC, there is a set of bands surrounded by light diffuse regions. The telomere region has a set of multiple thin bands.

Chromosome 2 is intermediate in length. The 2R arm has an asynaptic pericentromeric region without any remarkable bands. The telomere end is slightly flared and contains dark bands in region 6A-C. Two sets of dark bands in the middle of the arm in regions 9A and 9B are remarkable landmarks for the 2R arm. The 2L arm is similar to the 2R arm in length but could be distinguished from the 2R arm by the pericentromeric end, which is slightly asynaptic. The area of asynapsis in 2L is shorter than that in 2R and is restricted by a dark band in region 15A. The telomere end of the 2L arm has almost no bands in regions 20D-21A and looks significantly lighter compared with the dark-banded telomere of 2R arm.

Chromosome 3 is the longest in the chromosomal complement. The 3R arm has no asynapsis in the pericentric region, which is usually terminated by a short dark band with flare fibers in region 32C. The telomeric end of 3R is notably flared and marked by a wide light interband surrounded by two thin, dark bands in region 22B. Three bands in regions 28A, 28C, and in 29A can serve as additional landmarks for 3R. The 3L arm is the shortest autosomal arm. A long asynaptic region is located at the centromeric end of 3L. The telomeric end has no clear bands in region 39ED. The main landmark for 3L is a so-called “bird eye” landmark in region 36C. It is a dark, dot-shaped band, similar to a pupil, surrounded by a dark, long band, similar to an eyebrow.

### Physical mapping of orthologous genes to *An. beklemishevi* polytene chromosomes

**Fig. 3:**
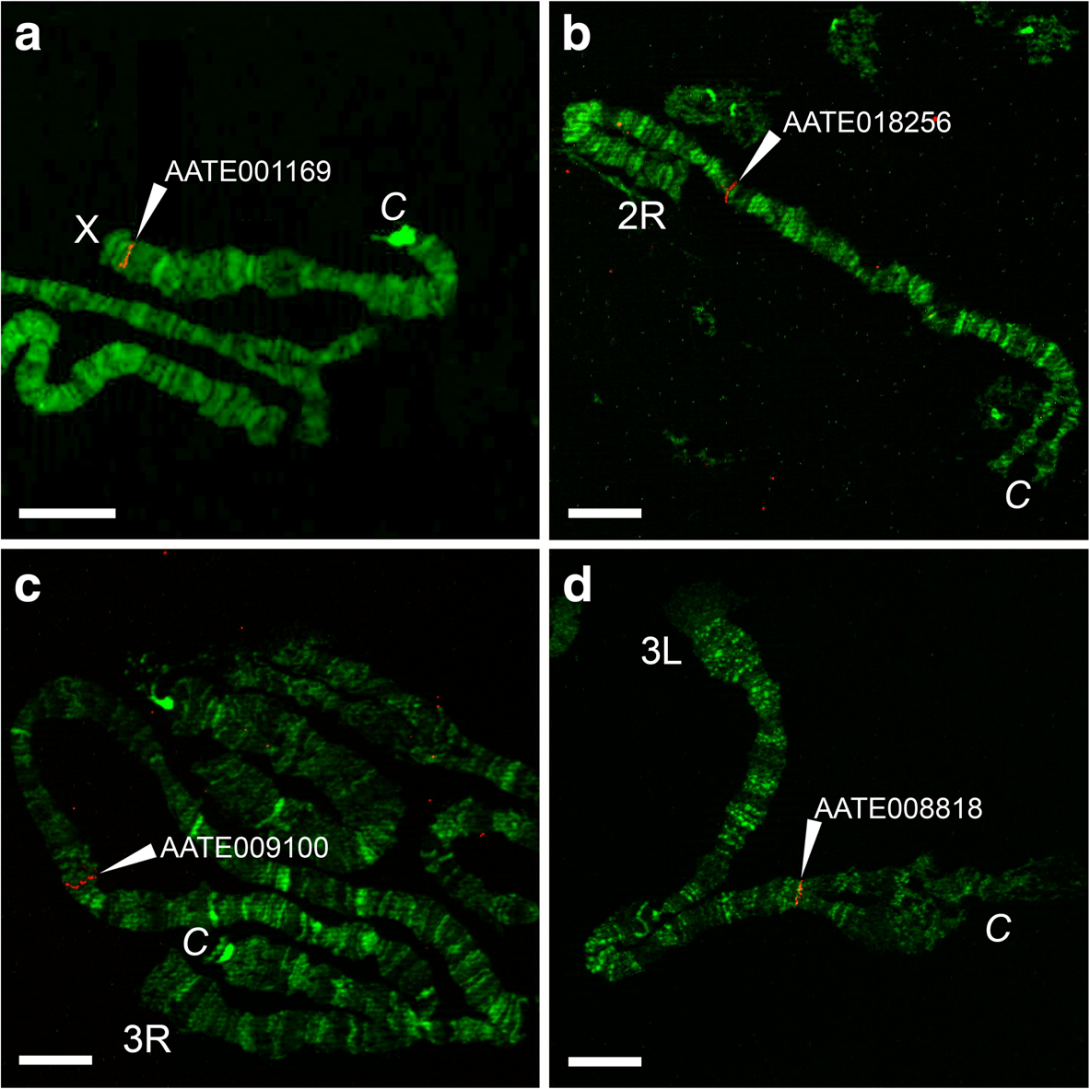
FISH on chromosomes of *An. beklemishevi*. The red signals (indicated by arrowheads) demonstrate the position of the orthologous *An. artroparvus* genes: AATE001169 (**a**), AATE018256 (**b**), AATE009100 (**c)** and AATE008818 (**d**) on *An. beklemishevi* chromosomes X, 2R, 3R and 3L, respectively. *Scale-bars*: 20 μm

To test the utility of the *An. beklemishevi* high-resolution chromosome map for physical gene mapping, we hybridized four orthologous genes of *An. atroparvus* to X, 2R, 3R, and 3L arms. Four PCR probes were designed to amplify the unique sequences from gene exons (Table 3). All probes produced clear and unique signals (Fig. 3) and were successfully mapped to the chromosome map based on the banding patterns (Fig. 2).

**Table 3:** The list of primer sequences for *An. atroparvus* genes used as PCR-based DNA markers to hybridize with chromosomes of *An. beklemishevi*

*An. atroparvus* gene	Chromosome	Forward primer	Reverse primer
AATE001169	X	TGGAGGACGTTCGGTTTCTG	AATTCCGACTGCTCGCTAGG
AATE017741	X	GCAGCTGTTATGTGCTTCGG	GTAGTACACCCAGTAGCGCC
AATE010870	X	CCAAATCTTCTCAATCGCCCG	CGTCAAGTCGTCCTCGGAAT
AATE018256	2R	AGTTGTGTACTGCATGGCGA	ACGATGGTCGCATAGAGCAG
AATE009100	3R	GGTGTGGTGCTGAAAATGTG	CACCTACACCCAGAGGCATT
AATE008818	3L	TCATGCTCGGCTGGTTCATT	CGTGAATGTGGTACGCAACG

**Fig. 4:**
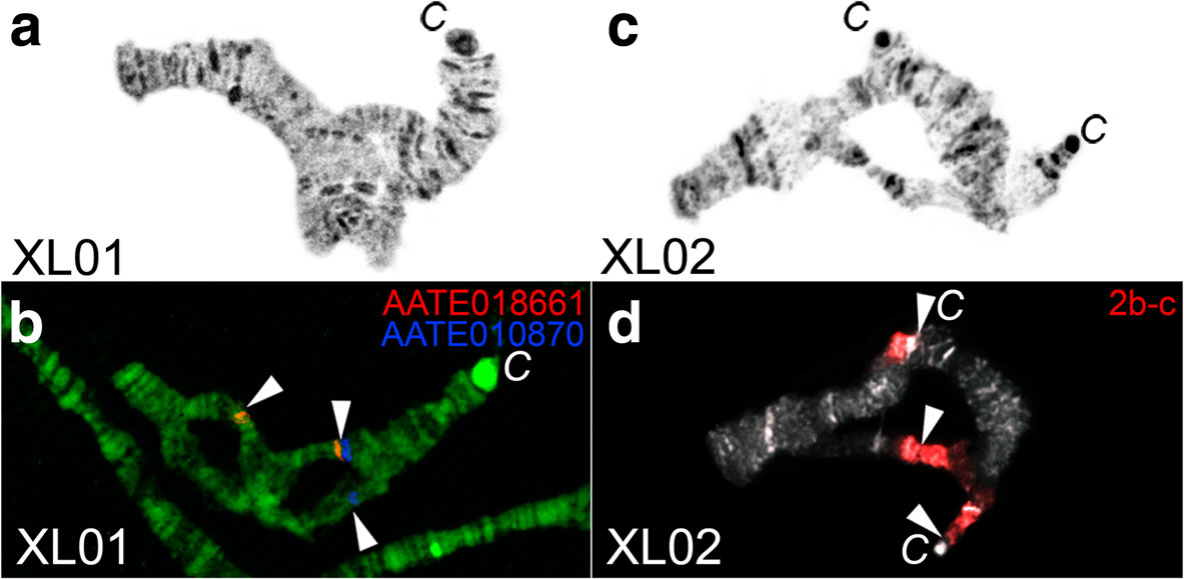
Physical mapping of the proximal breakpoint of X1 (**a**, **b**) and breakpoints of X2 (**c**, **d**) polymorphic inversions in *An. beklemishevi.* Phase contrast images of heterozygous X are shown on panels **a**, **c**, respectively. FISH of gene-specific DNA probes and micro-dissected DNA probes are demonstrated on panels **b**, **d**, respectively

To identify the exact cytogenetic positions of the proximal breakpoint of the X1 inversion, we mapped two DNA probes chosen from 24 genes mapped to *An. atroparvus* chromosomes [9]*.* The selected probes were expected to flank the proximal breakpoint of X1 inversion based on similarity of the banding pattern between the two species. Random primer-labelled DNA probes of genes AATE017741 and AATE010870 were hybridized to *An. beklemishevi* heterozygous chromosomes X01 that formed heterozygous loops (Fig. 4a). DNA probe AATE010870 was found in region 3C band 2 in both homologues, whereas juxtaposed gene AATE017741 was located in region 3B band 3 of one homologous chromosome and was found in region 1D of another homologue (Fig. 4b) indicating that the proximal breakpoint of this inversion is located between these two DNA probes.

To map the distal and proximal breakpoints of inversion X2, we micro-dissected the whole 2BC region of the X chromosome with the standard X00 arrangement. According to our cytogenetic analysis, this region crosses the distal breakpoint of X2 inversion. On heterozygous chromosome X02 (Fig. 4c), FISH of a micro-dissected DNA probe showed three signals (Fig. 4d). The longest signal marked entire region 2BC on the standard homologue. Another homologue had 2BC divided by the inversion into two fragments revealed by the probe as two signals. One of them was in region 2BC as standard, whereas the other was transposed to the pericentromeric region adjacent to region 5С band 3 where the other inversion breakpoint resides. Therefore, a single DNA probe FISH allowed us to map both breakpoints of the X2 inversion.

### Inversions in natural populations of *An. beklemishevi*

We analyzed inversion polymorphism in 18 collections from 13 Eurasian populations of *An. beklemishevi*. For two populations in Western Siberia, Kolarovo and Teguldet, collections were taken repeatedly during 3 and 4 years, respectively. For the inversion polymorphism analysis, we used both the original cytogenetic map for polytene chromosomes from salivary glands of larvae [8] and our newly developed map for the adult stage of mosquitoes. A total of 371 mosquitoes were analyzed at larva and adult stages. For the first time, 110 adult females were included in the inversion polymorphism analysis. Our data confirmed previous observations that *An. beklemishevi* is a polymorphic species [8]. Data on the inversion polymorphism are summarized in Table 1. In total, 4 of 12 well-known polymorphic inversions were found in 13 locations: X1, X2, 3R1 and 3R3. Inversion X1 was recorded in two distant locations of Karelia (Belomorsk) and Western Siberia (Chainsk). Inversion X2 is widespread from the White Sea to Siberia. Inversions 3R1 and 3R3 were detected only in a single population in Belomorsk.

All inversions, without exception, were found as heterozygotes only. Based on statistical analysis (Additional file 1: Table S1), none of the inversions displayed a significant (*P* < 0.05) departure from the Hardy-Weinberg equilibrium, suggesting that the absence of inverted arrangements of the chromosomal inversions is a result of the low frequency of inversions in the populations. However, a lethal effect of the inversion in a homozygote is possible and requires further experimental investigations.

## Discussion

We developed a high-resolution standard cytogenetic photomap for *An. beklemishevi*, which is one of the most northern species of the Maculipennis group and genus *Anopheles*. This map was developed using high-quality digital images similar to cytogenetic photomaps of other mosquito species [25, 29–34]. The goal of this work was to create a cytogenetic map that would be applicable for genome mapping and population genetics. In our study, the *An. beklemisevi* map was validated by mapping FISH signals of 4 PCR-amplified probes on X, 2R, 3R, and 3L chromosome arms. In addition, we mapped breakpoints on the X chromosome of *An. beklemishevi* using micro-dissected DNA probes. We demonstrated that the new cytogenetic map can serve as an effective tool for physical genome mapping of *An. beklemishevi*. Our study also confirmed the utility of the newly developed chromosome map for population genetic studies using adult females because this map was developed based on ovarian nurse chromosomes from the female mosquito. All population genetic studies conducted in the past included only the larval stage of mosquitoes because the map previously developed for *An. beklemishevi* was for the salivary gland chromosomes of fourth-instar larvae [8]. To our knowledge, inversion polymorphism in the adult stage of *An. beklemishevi* was studied for the first time here.

The analysis of inversion polymorphism in natural populations of *An. beklemishevi* demonstrated that chromosomal variations in this species are related to the geographical distribution of *An. beklemishevi*. This species inhabits the northern areas in the taiga zone. According to our data, the southern boundary of the species range is fragmented and located in the subzone of the southern taiga (the belt of mixed and broad-leaved forests). The northern boundary passes along the border of the taiga zone and forest tundra, near the 65th parallel in Europe and the 67th parallel in Asia [3, 35]. A high level of inversion polymorphism was noted in the subzone of the northern taiga: Belomorsk (Table 3), Syktyvkar and Berezovo [8]. Many inversions have also been identified in the adjacent area of the middle taiga: Petrozavodsk and Novaya Burka in the Tomsk region [8]. We propose that the central part of the taiga, which was formed since the end of the Neogene, provides optimal conditions for *An. beklemishevi* [36]. It should be expected that inversions of autosomes 2 and 3 will occur mainly in northern populations, where the species range does not overlap with the sibling species *An. messeae*. With the rising of global temperature, the *An. messeae* may displace *An. beklemishevi* in the subzone of the southern taiga. For example, a process of elimination of *An. beklemishevi* from the population has been demonstrated in Tomsk (South of Western Siberia). The proportion of this species in July samples has been reduced significantly from 0.109 in 1989 to 0.030 in 1998 [37]. The same process has been observed in two other locations in Western Siberia: Kolarovo and Cherga [3]. Reduction in the number of mosquitoes can lead to loss of chromosomal polymorphism in southern populations of *An. beklemishevi*. The effect of global warming has been shown for the population of another Palearctic member from the Maculipennis group, *An. maculipennis* [38].

In contrast to the autosomal rearrangements, inversions of chromosome X have a particular importance in the establishment of chromosomal polymorphism, especially the X2 inversion that is common throughout the species range (Table 3). Here we found no difference from the Hardy-Weinberg equilibrium for both autosomal and X chromosomal inversions. Nevertheless, overdominance of X2 was described in larvae from Shchuchye Lake which neighbors Teletskoe Lake (Gorniy Altay, Russia) [35]. Obviously, overdominance is the main way to maintain the X2 polymorphism; no homozygous X2 was detected, but heterozygotes dominated this population. Unlike X2, two homozygotes of X1 were identified in Syktyvkar [8]. The presence of common inversions may reflect the unity of the species population system throughout the range of *An. beklemishevi*.

## Conclusions

In this study, we developed a high-quality cytogenetic map for *An. beklemishevi,* a member of the Maculipennis group of malaria mosquitoes*.* Our study has demonstrated that a chromosomal photomap for *An. beklemishevi* can be utilized for physical genome mapping. We mapped to the chromosomes four random genes and the boundaries of two chromosomal inversions in chromosome X and indicated the positions of 12 other polymorphic inversions on the map. Polymorphic inversions were found in all parts of the species range. The highest level of chromosomal variations was noted North of the taiga zone. The analysis of the inversion polymorphism in natural populations of *An. beklemishevi* provides important insight into adaptive genetic variations of this species.

## Additional file

Additional file 1: Table S1. Chromosomal polymorphisms in populations of *Anopheles beklemishevi.* (DOCX 102 kb)
