# Supplementary material for: A standard photomap of ovarian nurse cell chromosomes and inversion polymorphism in Anopheles beklemishevi
Source: Parasit Vectors. 2018 Mar 27;11:211. doi: 10.1186/s13071-018-2657-3 (PMC5870207; doi:10.1186/s13071-018-2657-3)
Supplement: Supplementary file 1 — Chromosomal polymorphisms in populations of Anopheles beklemishevi. (DOCX 102 kb) [file 13071_2018_2657_MOESM1_ESM.docx]

Table S1. Chromosomal polymorphisms in populations of *An. beklemishevi*

| Localities (coordinates) | Collection date | Total (Female) number | Frequencies of inversions and significance level of Hardy-Weinberg deviation (p) | | | | | |
| --- | --- | --- | --- | --- | --- | --- | --- | --- |
|  |  |  | X1 | X2 | p | 3R1 | 3R3 | p |
| Kolarovo (56°20´N; 84°56´E) | 07/04/1983 | 23(8)L | 0 | 0.0625 | 0.99999993 | 0 | 0 | - |
| Kolarovo (56°20´N; 84°56´E) | 07/05/2004 | 6A | 0 | 0 | - | 0 | 0 | - |
| Kolarovo (56°20´N; 84°56´E) | 07/28/2005 | 2A | 0 | 0 | - | 0 | 0 | - |
| Teguldet (57°18´N; 88°10´E) | 08/05/2003 | 6A | 0 | 0 | - | 0 | 0 | - |
| Teguldet (57°18´N; 88°10´E) | 07/12/2005 | 27A | 0 | 0 | - | 0 | 0 | - |
| Teguldet (57°18´N; 88°10´E) | 07/13/2006 | 4A | 0 | 0.1111 | 0.99999844 | 0 | 0 | - |
| Teguldet (57°18´N; 88°10´E) | 08/01/2007 | 8A | 0 | 0 | - | 0 | 0 | - |
| Artybash (51°47´N; 87°15´E) | 07/26/2007 | 25(13)L | 0 | 0.2308 | 0.99989057 | 0 | 0 | - |
| Samysh River (51°45´N; 87°22´E) | 07/27/2007 | 14(9)L | 0 | 0.1667 | 0.99998434 | 0 | 0 | - |
| Koldor River (51°47´N; 87°43´E) | 07/28/2007 | 12(5)L | 0 | 0 | - | 0 | 0 | - |
| Kamga River (51°20´N; 87°47´E) | 07/29/2007 | 15(7)L | 0 | 0 | - | 0 | 0 | - |
| Dvorets (57°56´N; 32°60´E) | 06/01/2009 | 36(17)L | 0 | 0.3236 | 0.99906011 | 0 | 0 | - |
| Segezha (63°45´N; 34°46´E) | 08/16/2010 | 64(36)L | 0 | 0.1389 | 0.99840332 | 0 | 0 | - |
| Belomorsk (64°31´N; 34°46´E) | 08/14/2010 | 36(20)L | 0,0750 | 0.0500 | 0.99999698 | 0,0417 | 0.0139 | 0.99999997 |
| Dmitrovskiy Pogost (55°18´N; 39°50´E) | 07/23/2015 | 27(13)L | 0 | 0.1154 | 0.99999807 | 0 | 0 | - |
| Parykino (39°23´N; 55°17´E) | 07/24/2015 | 4(3)L | 0 | 0.3333 | 0.99885665 | 0 | 0 | - |
| Gzhel (55°36´N; 38°26´E) | 08/20/2015 | 5(4)L | 0 | 0.1250 | 0.99999698 | 0 | 0 | - |
| Chainsk (57°55´N; 82°36´E) | 06/22/2016 | 57A | 0.0088 | 0.1404 | 0.99999339 | 0 | 0 | - |

*Abbreviations*: L - larvae; A - adult females. Frequencies of inversion X1 and X2 were determined for females.
